# The mediating effect of psychological capital on the relationship between psychological stress and distress among chinese nursing students: a cross-sectional study

**DOI:** 10.1186/s12912-022-00915-0

**Published:** 2022-05-25

**Authors:** Feifei Sun, Aiqing Wang, Jiaomei Xue, Jing Su, Chuanfen Hu, Qinghua Lu

**Affiliations:** 1 Department of Clinical Psychology I, Shandong Mental Health Center, Shandong University, 49 Wenhua East Road, 250014 Jinan, Shandong China; 2 Department of Nursing, Shandong Mental Health Center, Shandong University, 49 Wenhua East Road, 250014 Jinan, Shandong China; 3 Society and Law School, Shandong Women’s University, No. 2399, University Road, Changqing University Science and Technology Park, Shandong 25030 Jinan, China; 4 Editorial Board, Journal of Shandong First Medical University, No. 6699 Qingdao Road, Huaiyin District, 250000 Jinan, China; 5 Department of Infection Management, Shandong Mental Health Center, Shandong University, 49 Wenhua East Road, 250014 Jinan, Shandong China

**Keywords:** Psychological capital, Perceived stress, Psychological distress, Mediation, Nursing students, China

## Abstract

**Background:**

With the COVID-19 outbreak in China, the Chinese government took measures to prevent and control the spread of the virus. In-person teaching was replaced by distance learning, which was an unknown challenge for students. In this context, little is known about the perceived distress of nursing students and the relationship between psychological capital, perceived distress, and psychological stress. This study examined the relationship between psychological capital, psychological distress, and perceived stress, and the mediating role of psychological capital in the relationship between perceived stress and psychological distress among nursing students.

**Methods:**

This cross-sectional survey was conducted between January and December 2020 using a convenience sampling method involving 359 undergraduate and specialist nursing students at a tertiary hospital in Shandong Province. Standardised instruments were used to measure psychological capital, psychological stress, and perceived stress. We used SPSS 24.0 and PROCESS macro to analyse the data.

**Results:**

There was a statistically significant difference in perceived stress among students based on whether they liked the nursing profession (*P* < 0.01). Relative to nursing college students, undergraduates experienced significantly higher levels of perceived stress (*P* < 0.01). Nevertheless, there were no significant differences in perceived stress according to gender, place of residence, and being an only child. Psychological distress was positively correlated (*r* = 0.632, *p* < 0.001) with perceived stress (*r *=-0.662, *p* < 0.001), whereas it was negatively correlated with psychological capital. Psychological capital played a potential mediating role in the relationship between psychological distress and perceived stress.

**Conclusions:**

Psychological distress was negatively correlated with psychological capital, and positively correlated with perceived stress. Mediation analyses indicated that psychological capital partially mediated the relationship between perceived stress and psychological distress. Educators should therefore heed students’ perceived stress and develop appropriate mental health counselling programmes for students in the curriculum that could help them reduce their psychological distress. In clinical practice, nursing managers must take effective measures, such as skills training, to improve the psychological capital of nursing students and reduce the negative impact of their psychological distress.

## Background

Stress is a complex psychobiological process that individuals experience when they perceive a threat or danger from their environment [1]. When individuals consider a situation threatening, they evaluate the resources available to address it. These assessments interact, leading to a perception of stress and the ensuing physical and emotional responses [2]. The topic of stress has drawn considerable attention in the literature and continues to be the subject of much research in the nursing field [3]. In December 2019, COVID-19 spread in China and subsequently worldwide [4]. Since April 2020, the Chinese government has implemented lockdown measures to prevent and control new coronavirus outbreaks in several regions, ceasing in-person teaching and switching to distance learning [5], which became a common stress source for many students [6]. Students’ fear of not being able to resume their normal lives due to the consequences of these quarantine measures, including reduced social contact, has resulted in increased stress levels [7].

Students do not initially consider education interruptions. With the additional pressures of educating nursing students for clinical practice, which includes the fear of making mistakes and having to deal with emergencies [8], this lack of applied skills, and the uncertainty around how to compensate for it, can also ad study phase, nursing students are chronically and heavily exposed to uncontrollable stressors that negatively impact their professional identity development and health[9].

Resilience appears to be a determinant of perceived stress [10]. Those with low emotional intelligence scores tend to adopt inappropriate coping strategies that are positively associated with maladaptive school behaviours and negatively associated with negative life events. Moreover, inappropriate coping regarding perceived stress may interfere with learning, decision-making, and thinking processes and lead to drowsiness, neurosis, or mental breakdowns that deteriorate professional competence [11].

Positive psychology has been receiving increasing attention. Some scholars have proposed a link between nursing students’ psychological capital and mental health improvements[12]. The introduction and application of psychological cap performance and goal achievement [13]. It has four main dimensions: self-efficacy, hope, resilience, and optimism [14]. In challenging academic environments, especially during the COVID-19 pandemic, students under greater pressure may require great self-efficacy to achieve task completion. Optimism helps students make positive attributions of success. Therefore, when problems and adversity arise, hope and resilience can be valuable psychological resources for persevering in achieving academic goals [15]. These dimensions may be regarded as positive states of psychological development exhibited by individuals in the growth and developmental process. Studies have shown that optimism and hope are statistically significantly negatively associated with psychological distress within the subcategory of psychological capital [16]. Previous research also shows that high levels of psychological capital weaken psychological distress [17]. Additionally, nursing students’ positive psychological capital has a significant role in helping them cope with stress and ensuring strong mental health [18]. Psychological capital and distress are closely related in connotation and extension [19].

Psychological distress is a common concern among nursing professionals [20]. It is known to be associated with various factors, such as occupations and geographical settings [21]. It ranges from normal feelings of vulnerability and sadness to mental health problems, such as depression, social isolation, and sleep disorders [22]. Nurses experience a wide range of psychosocial effects when caring for COVID-19 patients, including death anxiety, emotional distress caused by fear of contamination, the emergence of obsessive thoughts and fears, and interpersonal isolation[23]. This can exacerbate nurses’ psychological distress [24]. Psychological distress impacts job performance, overall life satisfaction, happiness, and the teamwork atmosphere [16]. One study in Italy found that more than 70% of nursing students experienced significant levels of psychological distress [25]. Nursing students may face traumatic events during their initial educational training; such experiences may lead to adverse psychological health outcomes, including anxiety, depression, and sleep disorders. Existing research indicates that negative mental health issues are a major cause of nursing student attrition [26].

The transition from nursing student to registered nurse is a critical time for nursing students and can be a stressful process [27]. It is thus important to focus on the COVID-19 pandemic’s psychological and professional impacts on nursing students, as this concerns their well-being and is vital to ensure the stability of the future nursing workforce. This study was undertaken because the increased perceived stress due to the pandemic can lead to an increased likelihood of negative health outcomes for nursing students, making it particularly important to understand the factors associated with their psychological distress. This study involved a specific assessment of stress in the clinical environment of nursing candidates who were transitioning from theoretical classes to the clinical practice phase, and therefore, fully considered the stress of the clinical environment for nursing students. Although a link between perceived stress and psychological distress has been evidenced [28], the mechanisms behind this link are not well understood by nursing students.

## Methods and measures

### Study aim

The objectives of this study are (1) to investigate the relationship between sociodemographic characteristics, such as age, gender, and education level, and nursing students’ perceived stress; (2) to examine the relationship between psychological stress, psychological capital, and perceived stress; and (3) to explore the role of psychological capital in mediating the relationship between psychological stress and perceived stress.

### Study design and data collection

This was a cross-sectional study comprising a descriptive survey. Nursing students were invited to complete an anonymous online survey between January and December 2020 during their clinical practice at Shandong Mental Health Center. Participants were recruited via a convenience sampling method. Using our sample size formula: *N *=(µαS/δ)2 (based on preliminary research, µα = 1.96, S/δ value = 9), and taking into account a 15% non-response rate, we decided on a minimum sample size of 357. A Total of 370 nursing students were recruited for the study, of which 359 qualified for the study criteria and completed the self-administered questionnaire, representing a response rate of 97.0%. The questionnaire took 15–20 min to complete. There were two main reasons for not responding to the study: some of the wards were too busy (eight students) and some were uninterested in the investigation (three students). Inclusion criteria included voluntary consent to participate in the study and undergraduate nursing students in the clinical practice phase. The exclusion criterion was students on rest or sick leave. The purpose and significance of the study were explained to the students through a face-to-face discussion, and their written consent for participation was obtained. The students then responded on a voluntary basis.

### Measures

#### Social-demographic questionnaire

Data were obtained using a self-designed questionnaire comprising questions regarding age, gender, education level, marital status, place of residence, being an only child, liking the nursing profession, and wanting to pursue nursing in the future. All items were self-evaluated.

#### Psychological capital questionnaire (PCQ)

We measured psychological capital using the Chinese version of the 24-item PCQ [14], which has four dimensions: self-efficacy, hope, resilience, and optimism.

Each of the four dimensions comprises six items, measured on a 6-point Likert scale, ranging from 1 (strongly disagree) to 6 (strongly agree). Sample items were as follows: ‘I feel confident and self-assured in my ability’ (self-efficacy); ‘I believe that I can accomplish my goal’ (hope); ‘I believe that I can bounce back from any setbacks that may occur’ (resiliency); and ‘I expect good things to happen in the future’ (optimism). This scale is widely used and displays good internal consistency. The reliability and validity of the scale are reported to be high, and it has been used in various Chinese studies [17]. In this study, the Cronbach’s alpha based on normalised terms for the scale was 0.903. Hotelling T-squared F = 1130.833. KMO sampling suitability number = 0.913. McDonald’s ω factor = 0.909.

#### Kessler psychological distress scale (K10) [29]

The Chinese version was translated and revised by the Institute of Social Medicine, Shandong University, with a Cronbach’s alpha of 0.80 and a reliability of 0.71, based on previous research [30]. The Chinese version of K10 comprises 10 items regarding the frequency of non-specific psychological distress. Sample questions included: ‘During the last 30 days, about how often did you feel tired out for no good reason?’ Each item is rated on a 5-point Likert scale, ranging from 1 (no time at all) to 5 (all the time). All items were scored, and the total score was categorised as either low (score 10–15), moderate (score 16–21), high (score 22–29), or very high (score 30–50). The scale is widely used and has good internal consistency. In our research, the Cronbach’s alpha based on normalised terms for the scale was 0.853. Hotelling T-squared F = 360.650, KMO sampling suitability number = 0.869. McDonald’s ω factor = 0.878.

#### Chinese perceived stress scale (CPSS) [31]

Yang et al. modified the perceived stress scale (PSS) into the Chinese version called the CPSS [32]. The scale has high reliability (Cronbach’s alpha = 0.81). It contains 14 items (e.g., ‘How often did you feel that you were unable to control important things in your life?’), with each item being measured using a 5-point Likert scale ranging from 1 (never) to 5 (always). Higher scores indicate more perceived stress symptoms. The total score was categorised into normal pressure (score 0–24), high pressure (score 25–42), excessive pressure (score 43–56), and very high pressure (score 30–50). The scale is widely used and displays good internal consistency. In the current study, the Cronbach’s alpha based on normalised terms for the scale was 0.839. Hotelling T-squared F = 316.204. KMO sampling suitability number = 0.870. McDonald’s ω factor = 0.878.

### Data analysis

SPSS 24.0 (IBM Corp., Armonk, NY, USA) were used for all data analysis.For testing the internal consistency of the measures, Cronbach’s alpha values were calculated at a significance level of *p* < 0.05. Descriptive statistics included mean, standard deviation (SD), number (n), and percentage. The independent variables t-test and one-way analysis of variance (ANOVA) were conducted to compare differences in perceived stress according to participants’ demographic characteristics. In addition, Pearson’s correlation analysis was performed to explore correlations between the variables of psychological distress, psychological capital, and perceived stress. Further, a hierarchical multiple regression analysis was used to determine the factors associated with perceived stress. The variance inflation factor (VIF) values of all predictive variables were less than 10, indicating negligible collinearity.

Hayes’ (2018) PROCESS macro for SPSS was used to test the mediating effects model [33]. It may be downloaded from the website processmacro.org [34]. It was considered significant if 95% confidence intervals (CI) did not include the value 0.

## Results

The sociodemographic characteristics (*N* = 369) and comparisons of perceived stress among nursing students are illustrated in Table 1. Participants’ mean age was 21.43 ± 0.751 years. Students who did not want to pursue nursing had significantly higher perceived stress scores than those who wanted to pursue nursing upon graduation (*P* < 0.01). There was a statistically significant difference in perceived stress among students based on whether they liked the nursing profession (*P* < 0.01). In comparison with college students, undergraduates had significantly higher levels of perceived stress (*P* < 0.01). However, there were no significant differences in perceived stress among the variables of gender, family residence, and being an only child.

**Table 1:** Sociodemographic characteristics of the participants

Variables	n (%)		M ± SD	T/F	P
Gender	Male	302 (84.1)	2.48 ± 0.49	1.690	0.194
	Female	57 (15.9)	2.38 ± 0.58		
Only child	Yes	85 (23.7)	2.37 ± 0.51	3.234	0.073
	No	274 (76.3)	2.49 ± 0.51		
Educational level	Undergraduate nursing students	356 (99.2)	2.46 ± 0.51	3.953	0.048
	specialist nursing students	3 (0.8)	1.88 ± 0.79		
Likes nursing profession	Like	157 (43.7)	2.35 ± 0.48	7.315	0.001
	Dislike	20 (5.6)	2.54 ± 0.52		
	Generally like	182 (50.7)	2.55 ± 0.51		
Whether you want to pursue nursing in the future	Yes	301 (83.8)	2.43 ± 0.51	7.080	0.008
	No	58 (16.2)	2.62 ± 0.48		
Place of residence	City	108 (30.1)	2.43 ± 0.51	0.517	0.473
	Countryside	251 (69.9)	2.47 ± 0.51		

The correlations between psychological distress, psychological capital, and each positive psychological capital domain are illustrated in Table 2

**Table 2:** Correlations between psychological capital, psychological distress, and perceived stress

	1	2	3
Psychological distress	1		
Psychological capital	− 0.410^**^	1	
Perceived stress	0.632^**^	− 0.662^**^	1

^**P < *662^

**Table 3:** Hierarchical linear regression analysis results

	Perceived Stress						
Variables	Step 1		Step 2		Step 3		
Gender	− 0.038	1.138	− 0.065	1.139	− 0.040	1.140	
Only child	0.072	1.349	− 0.032	1.370	− 0.060	1.373	
Age	0.609	1.010	0.439	1.013	0.231	1.020	
Educational level	0.086	1.109	0.048	1.124	− 0.008	1.186	
Likes nursing profession	0.116	1.116	0.095	1.117	0.129	1.120	
Whether you want to pursue nursing in the future	0.030	1.234	0.049	1.234	0.020	1.238	
Residence	− 0.038	1.138	0.567	1.038	0.400	1.218	
Psychological distress			− 0.065	1.139	− 0.491	1.296	
Psychological capital					− 0.040	1.140	
F	3.965**		37.102**		67.498**		
R2	0.063		0.425		0.607		
∆0.6	0.047		0.414		0.598		

^**P < *598^

Table 3 reveals the findings of the hierarchical linear regression analysis. During the first step, the direct effect of psychological distress on perceived stress (c-path) was verified after adjusting for covariates. In the second step, the mediating effect of psychological capital was validated. Asymptotic sampling and resampling strategies were applied to verify that psychological capital plays a potential mediating role in the relationship between psychological distress and perceived stress. Bootstrap estimation was based on 5,000 bootstrap samples. It was considered significant if 95% confidence intervals (CI) did not include the value 0.

The hierarchical multiple regression analyses were performed to explore the influential and mediating factors correlated with perceived stress. VIFs of all the independent variables were less than 10, which means that collinearity is not misleading. After age, gender, educational level, being an only child, liking nursing as a profession, wanting to pursue nursing after graduation, family residence, and shift patterns were adjusted for, psychological distress was negatively associated with perceived stress (β =-0.065, *P* < 0.01). Psychological distress accounted for 41.4% of the variance. Psychological capital was negatively associated with perceived stress (β =-0.040, *P* < 0.01), and psychological capital accounted for 59.8% of the variance in step 2. In the third step, the standardised regression coefficient (β) of psychological distress was reduced; therefore, psychological capital may have mediated the association between psychological distress and perceived stress.

**Table 4:** Effect of psychological distress on psychological capital

Psychological distress		Effect	*se*	*T*	*P*	*95fec LL–UL*	
Psychological capital	Total effect	0.5652	0.0377	14.9771	0.0000	0.4910–0.6394	
	Direct effect	0.3954	0.0340	11.6378	0.0000	0.3285–0.4622	
	Indirect effect	0.1699	0.0250			0.1216–0.2202	

*LLCI *Lower level for 95% confidence interval*. ULCI *Upper level for 95% confidence interval

The total effect of psychological distress on psychological capital (coeff = 0.5652, *p* = 0.000) and the direct effect (coeff = 0.3954, *p* = 0.000) were significant (Table 4). The indirect effect of psychological distress on perceived stress, mediated by psychological capital, was significant (95% confidence interval [CI] = 0.1216–0.2202).

## Discussion

This study examined the mediating effect of psychological capital on the relationship between psychological distress and perceived stress among nursing students. It demonstrated that psychological distress and psychological capital are directly related to perceived stress, and psychological capital is negatively correlated with perceived stress. The results of the mediating effect analysis showed that psychological capital is a partial mediator between psychological distress and perceived stress (95% CI = 0.1216–0.2202). This is consistent with previous findings that reported that psychological distress is significantly associated with perceived stress [35].

The results of this study have theoretical and clinical implications. They demonstrate that psychological distress is positively correlated with perceived stress among nursing students and align with previous studies [36]. Some studies have revealed that mental health conditions, such as depression, anxiety, and substance use disorders influence the level of perceived stress [37]. Furthermore, nursing students who struggle to come to terms with their emotions have poor emotional awareness, lack appropriate coping strategies, and experience higher levels of psychological distress [38]. During the COVID-19 outbreak, the Chinese government imposed a lockdown and home isolation of students, causing them considerable psychological stress [39]. Additionally, as China’s healthcare reform progresses, growing numbers of Chinese nurses are concerned about job stability, job rewards, and career prospects. This can subconsciously affect nursing students and their future career choices, resulting in higher levels of psychological stress [40].

Our study suggests that psychological capital is negatively associated with perceived stress among nursing students. Restrictions were introduced to minimise the spread of the virus. However, nursing students suffer from stress more often than students of other fields, and the more stress they are under, the more often they resort to avoidance behaviours or show helplessness [41]. This aligns with the findings of previous studies, which indicate that psychological factors have the greatest impact on stress levels [42]. One possible explanation for this finding is the role of the psychological characteristics of nursing students. People with positive psychological states perceive different levels of stress and, therefore, adopt different coping strategies. The high level of professional self-efficacy among nursing students may create an optimistic view of their future, enhance their sense of hope, and sharpen some of their positive coping strategies, thus, reducing the application of some poor coping methods [43]. An intervention study on perceived stress in informal cancer caregivers demonstrated that perceived stress was negatively associated with resilience and self-esteem. Research suggests that stress can be diminished by increasing the levels of resilience and self-esteem in caregivers [44].

Psychological capital assumed a significant mediating role in the current study. According to Siarava et al. [45], symptoms of psychological distress, particularly depression and anxiety, are associated with a lower quality of life. This can be explained by the fact that the performance of coping with emotional failure is motivated by actual or potential losses and failures. Such emotion regulation strategies and maladaptive coping mechanisms may occur over time, increasing the intensity of anxiety concerning adverse life events or tensions [46]. During the New Coronary Pneumonia epidemic, nursing students were inevitably affected by related negative information, resulting in negative emotions such as stress, anxiety and depression [47]. Since there is a link between psychological distress and the perception of negative events, the higher the individuals’ level of perception of negative events, the lower the level of their emotion regulation strategies [48] and the more their self-efficacy and self-esteem levels are challenged, affecting their resilience and increasing psychological distress. Likewise, the tendency to experience depression and negativity may be associated with a lower level of confidence in the resources available to them. This finding is significant considering the association between psychological capital and psychological distress. Alternatively, individuals with low levels of self-efficacy may have difficulty recognising their emotional experiences, such as narrative affective disorder, which may further lead to lower levels of self-reported psychological distress [49]. Psychological capital can help students cope with these negative effects and, according to previous studies, can be efficiently developed and managed [14]. The current findings support the role of psychological capital as a key mediating process that influences the degree of psychological distress in nursing students. Given the enormous importance of perceived stress on psychological distress—not to mention fluctuations in psychological capital—it seems that strengthening psychological capital at the individual level through interventions would be greatly beneficial.

We observed that nursing students’ pursuit of the nursing profession was closely related to perceived stress. Nursing students who liked the nursing profession experienced significantly less perceived pressure than those who did not, aligning with existing research results. Although the COVID-19 outbreak has caused nursing students to feel uneasy about their future, it has not dampened their interest in pursuing a career in nursing [50]. In addition, the perceived stress level of nursing students who wanted to pursue nursing was significantly lower than that of nursing students who did not want to pursue the profession after their graduation. This may be because nursing students feel that the profession aligns with their ambitions and skill set. They may identify with their field of study and have the necessary competencies to succeed [51].Only children have lower perceived stress scores than those of non-only children, a finding which is consistent with previous studies [52]. This may be because non-only children in China whose parents are not highly educated and have low socioeconomic status have many children and do not establish a close enough parent-child relationship with each child. In contrast, parents of an only child are often highly educated and have obvious socio-economic advantage and a closer parent-child relationship with their child. Due to the COVID-19 pandemic, parents have been separated from their families due to work and government lockdown measures. This consequently means that parent-child communication is limited, there is a lack of family understanding and support, and the social support system for non-only children is inadequate [53]. Therefore, the perceived stress levels of non-only children were higher than those of only children. In this study, no gender differences were statistically significant in terms of perceived stress. Consistent with other studies [54], the higher perceived stress scores of males than females in this study may be due to changes in the learning environment, lower socioeconomic levels, lack of communication with friends, family role conflicts, and pressure due to a lack of preparation for employment caused by the pandemic. Place of residence was not statistically significant in terms of perceived stress. Similar to other studies [55], the current results indicate that the correlation between the place of residence and perceived stress is not significant.

There are several limitations to this study. First, since the study had a cross-sectional design, it could only examine the causal relationships between variables. Further longitudinal studies are needed to infer causality. Second, the sample size of this study was small; this limits the applicability of the results and weakens the statistical power. Longitudinal empirical studies with large samples are required to determine causal relationships and the differences between variables. Third, all data collected were through self-reported questionnaires, which inevitably produces reporting bias. This study is quantitative; a qualitative exploration is necessary to ensure a deeper understanding of the influence of psychological capital among nursing students.

## Conclusions

Overall, psychological distress and psychological capital are directly related to perceived stress, and psychological capital is negatively correlated with perceived stress. The results of the mediating effect analysis showed that psychological capital is a partial mediator between psychological distress and perceived stress. Educators should therefore heed students’ perceived stress and develop appropriate mental health counselling programmes for students in the curriculum that could help them reduce their psychological distress. In clinical practice, nursing managers must take effective measures, such as skills training, to improve the psychological capital of nursing students and reduce the negative impact of their psychological distress.

The psychological capital intervention (PCI) model [56] provides guidelines to develop self-efficacy, hope, resilience, and optimism. Through PCI training, self-efficacy in nursing students may be ameliorated, further allowing them to experience success and realise their targets through partner encouragement. Resilience may be enhanced by encouraging nursing students to exercise their ability to forecast and manage setbacks related to personal goal setting or other work events. Positive meditation can be offered to reduce stress and increase positive thinking in nursing students who are experiencing stress. Accordingly, our findings provide important insights that can be applied to future research on mental health resources for nursing students [57].

## Data Availability

Due to IRB protocols, the datasets generated and analysed for the current study are not publicly available but are available from the first author upon reasonable request.

## Abbreviations

PCQ: Psychological capital questionnaire
K10: Kessler psychological distress scale
CPSS: Chinese perceived stress scale
PCI: Psychological capital intervention
VIF: Variance inflation factor
